# Perforation d’anévrysme de la valve mitrale postérieure: complication rare de l’endocardite infectieuse: à propos d’un cas

**DOI:** 10.11604/pamj.2019.32.178.17710

**Published:** 2019-04-11

**Authors:** Loubna Hara, Fatima-Zahra El Hattab, Fatima-Zohra Radi, Jamila Zarzur, Mohamed Cherti

**Affiliations:** 1 Service de Cardiologie B, CHU Ibn Sina, Rabat, Maroc

**Keywords:** Anévrysme mycotique, valve mitrale postérieure, endocardite infectieuse, insuffisance aortique, ETT, ETO, Mycotic aneurysm, posterior mitral valve, infectious endocarditis, aortic regurgitation, TTE, TEE

## Abstract

L'anévrysme de la valve mitrale est une anomalie rare dont la physiopathologie est mal élucidée. Il se définit par une protubérance localisée au niveau du feuillet valvulaire mitral bombant dans l'oreillette gauche. La localisation sur le feuillet postérieur est exceptionnelle. Nous rapportons le cas d'un jeune homme de 26 ans suivi depuis 4 ans pour une insuffisance aortique rhumatismale qui est hospitalisé pour un syndrome fébrile avec poussée d'insuffisance cardiaque gauche. L'échocardiographique transthoracique (ETT) et transoesophagienne (ETO) ont mis en évidence des végétations sur la valve aortique avec un large anévrysme de la petite valve mitrale associé à une fuite mitrale importante. Le patient a bénéficié d'un double remplacement valvulaire mitral et aortique avec des suites opératoires simples. Une suspicion clinique avec une imagerie adaptée préopératoire et un traitement chirurgical à temps sont nécessaires pour reconnaître et traiter cette complication rare de l'endocardite infectieuse.

## Abstract (en)

Mitral valve aneurysm is a rare abnormality whose pathophysiology is poorly understood. It is defined as a bulge of the mitral valve leaflet toward the left atrium. Aneurysm in the posterior leaflet is exceptional. We report the case of a 26-year old man, who had been followed up for rheumatic aortic regurgitation for 4-years, hospitalized for febrile syndrome associated with episodes of left heart failure. Transthoracic echocardiographic examination (TTE) and transesophageal echocardiography (TEE) showed aortic valve vegetations with wide aneurysm of the small mitral valve associated with severe mitral valve regurgitation. The patient underwent mitral and aortic valve replacement with simple postoperative outcome. Clinical suspicion associated with suitable preoperative imaging and early surgical treatment are essential to recognize and treat this rare complication of infectious endocard

## Introduction

L'anévrysme de la valve mitrale postérieure est une entité bien définie, mais reste une anomalie très rare dont la physiopathologie est mal élucidée. Nous présentons un cas d'anévrysme de la valve mitrale postérieure dans un contexte d'endocardite infectieuse mitro-aortique.

## Patient et observation

**Figure 1:**
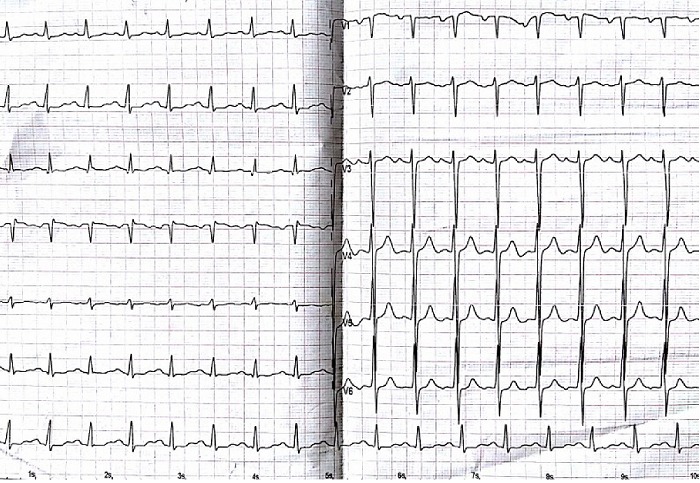
Electrocardiogram (ECG) montrant un rythme régulier sinusal avec une hypertrophie auriculaire et ventriculaire gauche

**Figure 2:**
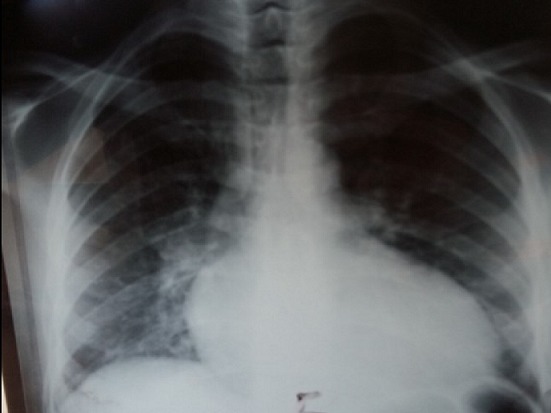
Radiographie pulmonaire de face montrant une cardiomégalie par allongement de l’arc inférieur gauche, un débord droit et une surcharge hilaire bilatérale

**Figure 3:**
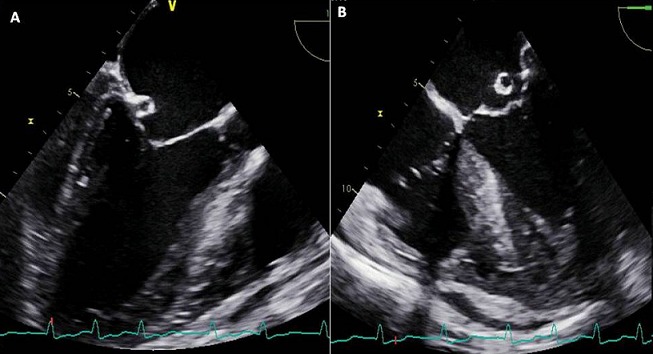
Coupe trois cavités et quatre cavités en échographie transoesophagienne (ETO) montrant une formation sacciforme au niveau du versant auriculaire de la valve mitrale postérieure

**Figure 4:**
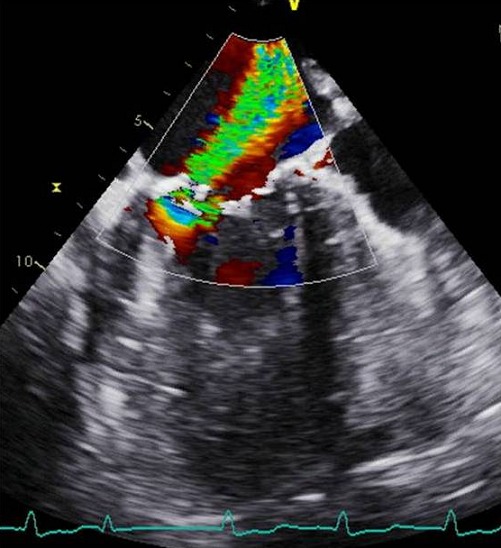
Coupe 3 cavités en échographie transoesophagienne (ETO) au Doppler couleur montrant une fuite mitrale importante à travers l’anévrysme mycotique

**Figure 5:**
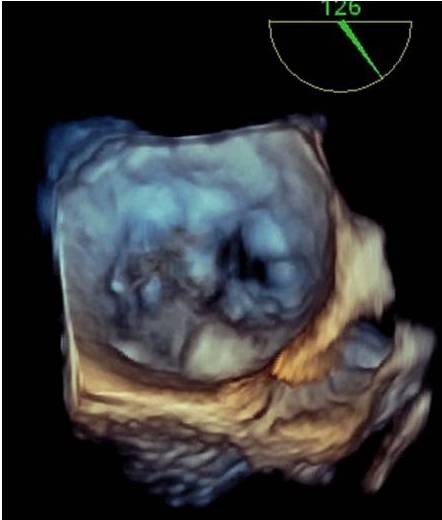
Echocardiographie tridimensionnelle en échographie transoesophagienne (ETO) misant en évidence l’anévrysme mycotique de la valve mitrale postérieure

Mr AM âgé de 26 ans est connu porteur d'une insuffisance aortique rhumatismale depuis 4 ans initialement modérée, devenant importante au cours du suivi. Le patient était asymptomatique durant ces années. Un mois avant son hospitalisation, il rapporte une dyspnée au moindre effort avec altération de l'état général et fébricule. L'examen physique à l'admission trouve un patient fébrile à 38,8°C, polypnéique (24 cpm), pâle, cachectique, tachycarde à 120 bpm avec une tension artérielle (TA) à 100/40 mmHg, une SpO2 à 92% à l'air ambiant et des signes périphériques d'insuffisance aortique. L'auscultation cardiaque trouve un souffle diastolique en latérosternal gauche accompagné d'un souffle systolique apexien irradiant vers l'aisselle gauche d'apparition récente. L'examen pleuropulmonaire note des râles crépitants bilatéraux arrivant à mi-champs. L'électrocardiogramme inscrit une tachycardie sinusale à 106 bpm et une hypertrophie ventriculaire et auriculaire gauche Figure 1. À la radiographie du thorax, une cardiomégalie par allongement de l'arc inférieur gauche, un débord droit et une surcharge hilaire bilatérale étaient notés Figure 2. La biologie a mis en évidence une hyperleucocytose à 12.700 et un syndrome inflammatoire avec une vitesse de sédimentation accélérée à 58 mm la première heure et une protéine C réactive à 118 mg/l. L'échographie cardiaque transthoracique et transoesophagienne ont montré des valves aortiques remaniées épaissies avec une fuite aortique importante et un magma de végétations sur les cusps, la valve mitrale était fine mais siège au niveau de son feuillet postérieur d'une formation sacciforme anévrysmale faisant 10 mm de grand axe, bombant dans l'oreillette gauche et communiquant avec le ventricule gauche Figure 3. L'anévrysme était perforé, ce qui donnait au doppler couleur une fuite mitrale importante se faisant uniquement à travers la perforation anévrysmale Figure 4. L'échocardiographie tridimensionnelle a permis une meilleure description de l'anévrysme Figure 5. Le ventricule gauche est modérément dilaté et sa fonction systolique est conservée. L'oreillette gauche est légèrement dilatée. Le diagnostic d'une endocardite infectieuse est retenu. Après une série d'hémocultures, le patient est mis sous traitement antibiotique: ampicilline en association avec la gentamycine. La chirurgie urgente est indiquée. Le patient est opéré après stabilisation de son état respiratoire et hémodynamique. L'exploration opératoire a mis en évidence un anévrysme de la petite valve mitrale avec perforation centrale. L'intervention a consisté en un double remplacement valvulaire mitro-aortique par deux valves mécaniques. Les suites opératoires étaient simples. L'antibiothérapie a été maintenue pendant six semaines. L'évolution a été marquée par l'apyrexie et la disparition du syndrome inflammatoire biologique. L’échographie cardiaque transthoracique (ETT) postopératoire trouve deux prothèses mitro-aortique de bon fonctionnement.

## Discussion

L'anévrysme de la valve mitrale est une entité très rare, il est fréquemment associé à une endocardite infectieuse aortique [1]. Son incidence est de l'ordre de 9,6% lors d'une endocardite du cœur gauche. Le premier cas est décrit dans la littérature par Morand en 1729 [2]. Depuis, plusieurs cas ont été rapportés sporadiquement. L'anévrysme de la valve mitrale se définit par une protubérance localisée au niveau du feuillet valvulaire mitral bombant dans l'oreillette gauche, elle s'expant en systole et elle se collabe en diastole [3]. D'après les cas rapportés dans la littérature ainsi que dans une série de Vilacosta de 13 cas d'anévrysme de la valve mitrale publiée en 1999 [3], l'atteinte anévrysmale des valves touche exclusivement la mitrale, en particulier son feuillet antérieur; de rares cas d'anévrysme du feuillet postérieur ont été rapportés [4, 5]. L'anévrysme de la valve mitrale est souvent associé à une endocardite infectieuse. Les rares cas en dehors de ce contexte ont une des pathologies suivantes: connectivites, dégénérescence valvulaire myxœdémateuse, pseudoxanthome élastique ou lors d'une insuffisance aortique sévère [6]. Dans notre cas, le patient a une endocardite infectieuse certaine aortique et mitrale. Le mécanisme exact de la formation de cet anévrysme mitral est encore mal élucidé. L'hypothèse la plus plausible est que l'infection est responsable d'une inflammation et d'une fragilisation du tissu valvulaire causant ainsi avec les forces mécaniques du jet de régurgitation aortique la formation d'une poche anévrysmale du feuillet mitral en question [7]. Pour notre patient, il présente une insuffisance aortique importante, qui s'est compliquée d'une endocardite infectieuse. L'anévrysme de la valve mitrale peut se compliquer par une rupture avec comme conséquence une fuite mitrale. L'autre complication possible est thromboembolique avec possibilité de formation de thrombus au fond de l'anévrysme. Chez notre patient l'anévrysme s'est rompu, donnant ainsi une insuffisance mitrale aiguë responsable du tableau d'insuffisance cardiaque gauche.

L'échographie cardiaque transthoracique est l'examen le plus pratiqué en routine, il est à faire en première intention. Mais l'échographie transoesophagienne (ETO) est beaucoup plus sensible en matière de détection d'anévrysme de la valve mitrale et surtout en ETO tridimensionnelle [8]. Dans notre cas, le diagnostic a été établi grâce à l'échographie transthoracique et l'ETO n'a fait que le confirmer. Les diagnostics différentiels à évoquer devant l'aspect échographique sont: le prolapsus de la valve mitrale, le myxome de la valve mitrale, le kyste de la valve mitrale sans endothélisation ou le diverticule (congénital) de la valve mitrale. L'analyse soigneuse en bidimensionnel, ainsi qu'en doppler couleur, aide à faire la distinction entre l'anévrysme et les autres anomalies, en démontrant la communication directe entre l'anévrysme et le ventricule gauche. Le traitement de l'anévrysme de la valve mitrale n'est pas bien défini. Malgré l'enthousiasme de certains auteurs pour le traitement médical, l'attitude la plus communément admise reste chirurgicale du fait des complications de ces anévrysmes. La chirurgie réparatrice peut être envisagée. Cette réparation de la valve mitrale se fait souvent par un patch autologue péricardique. La qualité du geste doit être contrôlée par l'échographie transoesophagienne peropératoire [3, 4]. Un cas a été décrit dans la littérature [9] où la réparation de l'anévrysme de la valve mitrale est faite par des points de suture directs par un fil en prolène 4.0. Le patient a récidivé son anévrysme au bout de quatre mois et il a alors bénéficié d'un remplacement valvulaire mitral par une bioprothèse. Le remplacement de la valve mitrale est indiqué dans les situations où l'anévrysme est large ou lorsqu'il y a une rupture anévrysmale avec un appareil sous-valvulaire détruit ainsi qu'en cas de remplacement valvulaire aortique associé [3, 4]. D'après la série de Vilacosta *et al.* [3], le traitement chirurgical est indiqué dans 8 cas sur 13 et le remplacement valvulaire mitral est pratiqué dans tous les cas. Dans notre observation, on a décidé de remplacer la valve mitrale vu la nécessité de réaliser un remplacement valvulaire aortique et surtout que dans notre contexte on n'a pas d'ETO peropératoire qui nous permet de réaliser une bonne plastie mitrale. L'équipe a préféré la sécurité d'un double remplacement valvulaire mitro-aortique afin d'éviter une éventuelle chirurgie tridux ultérieure.

## Conclusion

L'anévrysme de la valve mitrale est une entité rare. Sa localisation sur le feuillet mitral antérieur est la plus fréquente, la localisation postérieure est exceptionnelle. L'association à une endocardite aortique est classique. Son traitement peut être conservateur par plastie mitrale mais, en cas de nécessité d'un remplacement valvulaire aortique, un remplacement de la valve mitrale peut être nécessaire.

## Conflits d’intérêts

Les auteurs ne déclarent aucun conflit d'intérêts.
